# Microsatellite analysis and polymorphic marker development based on the full-length transcriptome of *Camellia chekiangoleosa*

**DOI:** 10.1038/s41598-022-23333-3

**Published:** 2022-11-07

**Authors:** Qianqian Tian, Bin Huang, Jianjian Huang, Bo Wang, Le Dong, Xin Yin, Chun Gong, Qiang Wen

**Affiliations:** 1 Jiangxi Provincial Key Laboratory of Camellia Germplasm Conservation and Utilization, Jiangxi Academy of Forestry, Nanchang, 330047 China; 2 College of Forestry, Jiangxi Agricultural University, Nanchang, 330045 China

**Keywords:** Plant genetics, Sequencing, Genetics, Molecular biology, Transcriptomics

## Abstract

*Camellia chekiangoleosa* is a popular variety of Oil-camellia that has high oil production and ornamental value. Microsatellite (SSR) markers are the preferred tool for the molecular marker-assisted breeding of *C. chekiangoleosa*. By focusing on the problems of the low development efficiency of polymorphic SSR markers and the lack of available functional markers in Oil-camellia, we identified 97,510 SSR loci based on the full-length transcriptome sequence of *C. chekiangoleosa*. An analysis of SSR characteristics showed that mononucleotide (51.29%) and dinucleotide (34.36%) SSRs were the main repeat types. The main SSR distribution areas based on proportion covered were ordered as follows: 5'UTR > 3'UTR > CDS. By comparing our data with those in databases such as GO and KEGG, we obtained functional annotations of unigene sequences containing SSR sites. The data showed that the amplification efficiency of the SSR primers was 51.72%, and the development efficiency of polymorphic SSR primers was 26.72%. Experiments verified that dinucleotide and pentanucleotide SSRs located in UTR regions could produce more polymorphic markers. An investigation into the genetic diversity of several *C. chekiangoleosa* populations also suggested that the developed SSR markers had higher levels of polymorphism. This study will provide a reference and high-quality markers for the large-scale development of functional SSR markers and genetic research in Oil-camellia.

## Introduction

Oil-camellia is the common name for woody oil trees of the *Camellia* genus of the family Theaceae that have high oil contents and economic value. *C. oleifera* is a well-known species in this family^1^. *C. chekiangoleosa* is an endemic variety of Oil-camellia in China. Its oil is rich in unsaturated fatty acids, and its seed oil content and oil quality are greater than those of *C. oleifera*^2^. In addition to its high oil-producing value, *C. chekiangoleosa* has high ornamental value and is an important garden tree species^3^. *C. chekiangoleosa* is a typical diploid tree species and does not show compound polyploidy^4,5^. It is naturally distributed in the mountains of Jiangxi, Zhejiang and northern Fujian at an altitude of 600–1400 meters^4^. The unique traits of this species are obviously distinguishable in the forest, and it presents rich genetic variation. To date, no varieties or superior clones of this species have been developed^6–10^, and there is an urgent need to accelerate the breeding of *C. chekiangoleosa*.

Molecular marker-assisted selection (MAS) can be employed to accurately estimate genetic backgrounds, quickly screen target traits, improve breeding efficiency, and shorten the breeding cycle^11^. Microsatellite markers currently have great advantages compared with other molecular marker technologies, including high versatility, high polymorphism, codominance, stability and reliability^12,13^. Microsatellites, also called simple sequence repeats (SSRs), are tandemly repeated DNA sequences with basic units of 1 to 6 nucleotides that are widely distributed throughout eukaryote genomes^14,15^. The sequences employed for SSR marker development have traditionally come from constructed gene libraries or shared sequences in public gene databases (NCBI, EMBL, and DDBJ)^16^. The disadvantages of this approach include low development efficiency and limited gene sequence resources, making it difficult to apply to nonmodel organisms^16^. With the maturation of high-throughput sequencing technology, the use of sequencing to obtain a large number of DNA sequences to develop SSR markers has gradually become the mainstream method. In recent years, there have been many reports on SSR marker-related research in *C. oleifera*. The initial data employed for marker development mainly came from parts of the genome and transcriptome obtained by first- and second-generation sequencing technologies and limited expressed sequence tags (ESTs)^17–19^. In contrast to the studies conducted in *C. oleifera*, there are relatively few reports on SSR markers in *C. chekiangoleosa*, and few markers are available for this species. Wen et al. analyzed the composition and distribution characteristics of SSR sequences in the *C. chekiangoleosa* transcriptome based on 454 sequencing, and 18 polymorphic SSR markers were developed in subsequent research^20,21^. Shi et al. used the same sequencing technology to develop 109 SSR markers based on a partial genome sequence of *C. chekiangoleosa*^22^. The existing analyses show that the SSR markers available for the future molecular selection-based breeding of *C. chekiangoleosa* are far from sufficient; in particular, functional SSR molecular markers closely related to target traits have yet to be developed.

Full-length transcriptome sequencing (Iso-Seq, isoform sequencing) based on third-generation sequencing technology presents the advantages of ultralong read lengths, no template amplification, low time consumption, gene family characterization, and more comprehensive and accurate sequencing results and shows great advantages in identifying homologous genes, transcripts of superfamily genes, allele expression and transcription annotations^20–22^. However, third-generation sequencing technology also has some shortcomings, such as generating inaccurate genetic information, but the correction of transcripts by second-generation sequencing technology can compensate for this deficiency^23,24^. EST-SSR markers may be directly related to functional genes, and their development efficiency is higher than that of genomic SSRs (g-SSRs)^25^. Therefore, with the increasing emphasis on functional genomics, it is possible to use the abundant sequence resources provided by full-length transcriptome data and information such as functional annotation and transcription factor (TF) prediction to develop functional SSR markers on a large scale. Research on the development of SSR markers based on full-length transcriptome data has been reported in a variety of plants. For example, Wu et al. identified 23,239 SSRs in 42,323 *Populus wulianensis* transcript sequences, designed 100 EST-SSR markers for verification, and finally obtained 88 qualified markers, 18 of which were polymorphic^26^. Xiao et al. obtained 847 (79.16%) amplifiable markers from 1070 sugarcane EST-SSR markers, including 349 (32.60%) that were polymorphic^27^. These studies show that it is feasible to develop SSR markers using full-length transcriptome sequences. However, there are no reports of the use of full-length transcriptomes to develop SSR markers in Oil-camellia.

Based on the abundant sequence resources provided by the full-length transcriptome data of *C. chekiangoleosa* (NCBI accession number PRJNA753883), which were corrected by the second-generation data^28^, this study analyzed the distribution characteristics of all SSR sites among full-length transcripts, quantified their functional annotations and obtained TF information for the full-length transcripts containing SSRs. We assessed the SSR development efficiency and analyzed the polymorphic SSR ratio of dinucleotide, trinucleotide, tetranucleotide and pentanucleotide repeats, as well as SSRs located in coding regions (CDS) and noncoding regions (UTR), and we carried out large-scale development of SSR markers and the detection of some polymorphic SSR markers using population genetic investigation. Our research not only enriches the available information on the distribution of SSR loci among the expressed sequences of *C. chekiangoleosa* but also provides a large number of functional marker resources for MAS of *C. chekiangoleosa*. At the same time, this study provides a valuable method to efficiently develop SSR markers for other Oil-camellia species.

## Results

### Identification and characterization of SSRs in the transcriptome

Microsatellites can be divided into perfect SSRs, imperfect SSRs and composite SSRs^29^. In this study, the perfect and composite SSRs in the full-length transcriptome of *C. chekiangoleosa* were statistically analyzed, and some microsatellite information is shown in Table 1. A total of 97,510 SSRs (including 17,690 composite SSRs) were retrieved from 65,215 unigene sequences with a total length of 188,333,521 bp, among which 48,281 unigene sequences contained SSRs. The frequency of the occurrence of SSRs was 74.03%, with an average of 1 SSR occurring every 1.93 kb. There were significant differences in the frequency of each SSR repeat type in the full-length transcriptome of *C. chekiangoleosa*. Mononucleotide repeats were the main repeat type, accounting for 51.29% of the total SSRs, followed by dinucleotide (34.36%), trinucleotide (11.24%), tetranucleotide (1.44%), hexanucleotide (1.11%) and pentanucleotide repeats (0.56%) (Table 1).

**Table 1:** The number and frequency of SSRs in *C. chekiangoleosa.*

Characters	Transcript sequence
Total number of sequences examined	65,215
Total size covered by examined sequences/bp	188,333,521
Total number of SSRs identified	97,510
Number of compound microsatellites	17,690
Number of SSR-containing sequences	48,281
Total frequency of occurrence	0.74
Average distance/bp	1931.43
Mononucleotide repeat (MNRs)	40,942 (51.29%)
Dinucleotide repeat (DNRs)	27,428 (34.36%)
Trinucleotide repeat (TNRs)	8974 (11.24%)
Tetranucleotide repeat (TTNRs)	1146 (1.44%)
Pentanucleotide repeat (PTNRs)	442 (0.56%)
Hexanucleotide repeat (HXNRs)	889 (1.11%)

According to the motifs of several of the main SSR repeat types (Supplementary Table S1), there were 2, 4, 12 and 30 motifs for the mono-, di-, tri- and tetranucleotide repeat types, respectively. Mononucleotide repeats were dominated by A/T repeat motifs, accounting for 49.95% of these repeats, while the number of C/G repeat motifs was relatively small, accounting for 1.34% of these repeats. Among dinucleotide repeats, the number of AG repeats (23.66%) was highest, followed by AT (7.89%) and AC repeats (2.67%), while the number of CG repeats (0.04%) was lowest. Among the trinucleotide repeats, AAG (2.29%) repeats accounted for the largest proportion, followed by ATT (1.28%) and ACC (1.83%), and the proportions of the other nine repeat motifs were all low. Among the tetranucleotide repeats, A/T-rich repeat motifs (AAAT, AAAG, AAAC, AACT, AATC, AATG, AATT, AGAT, ATAC, ATTT) accounted for 1.24% of all SSR repeat types, while G/C-rich motifs were relatively rare. We found that all the major repeat motifs of different SSR repeat types were rich in A/T nucleotides.

**Figure 1:**
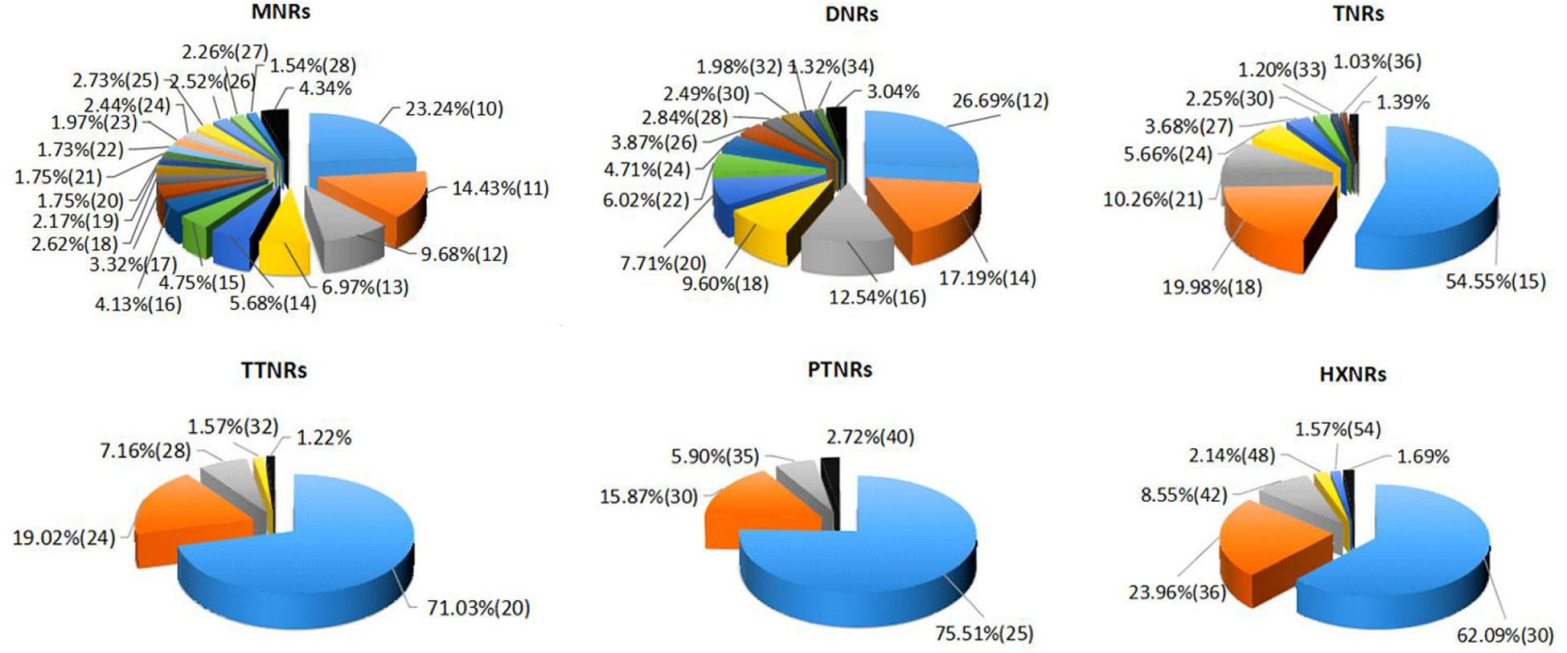
Lengths of different types of microsatellites. Each section of the pie chart corresponds to SSRs of the same length. If the corresponding SSR length frequency is less than or equal to 0.01, the SSRs are merged together in the black section.

There were significant differences in the length variation of different repeat types of SSRs in the whole transcriptome of *C. chekiangoleosa* (Fig. 1). The number of sections in the pie chart represents the variation in SSR length. The more sections there are, the higher the polymorphism of the SSRs. Based on the changes in the number of sections, the highest degree of length variation was found for mononucleotide repeats, while the lowest was found for pentanucleotide repeats. For mononucleotide to pentanucleotide repeats, the variation in the SSRs was inversely proportional to the length of the repeat type.

**Figure 2:**
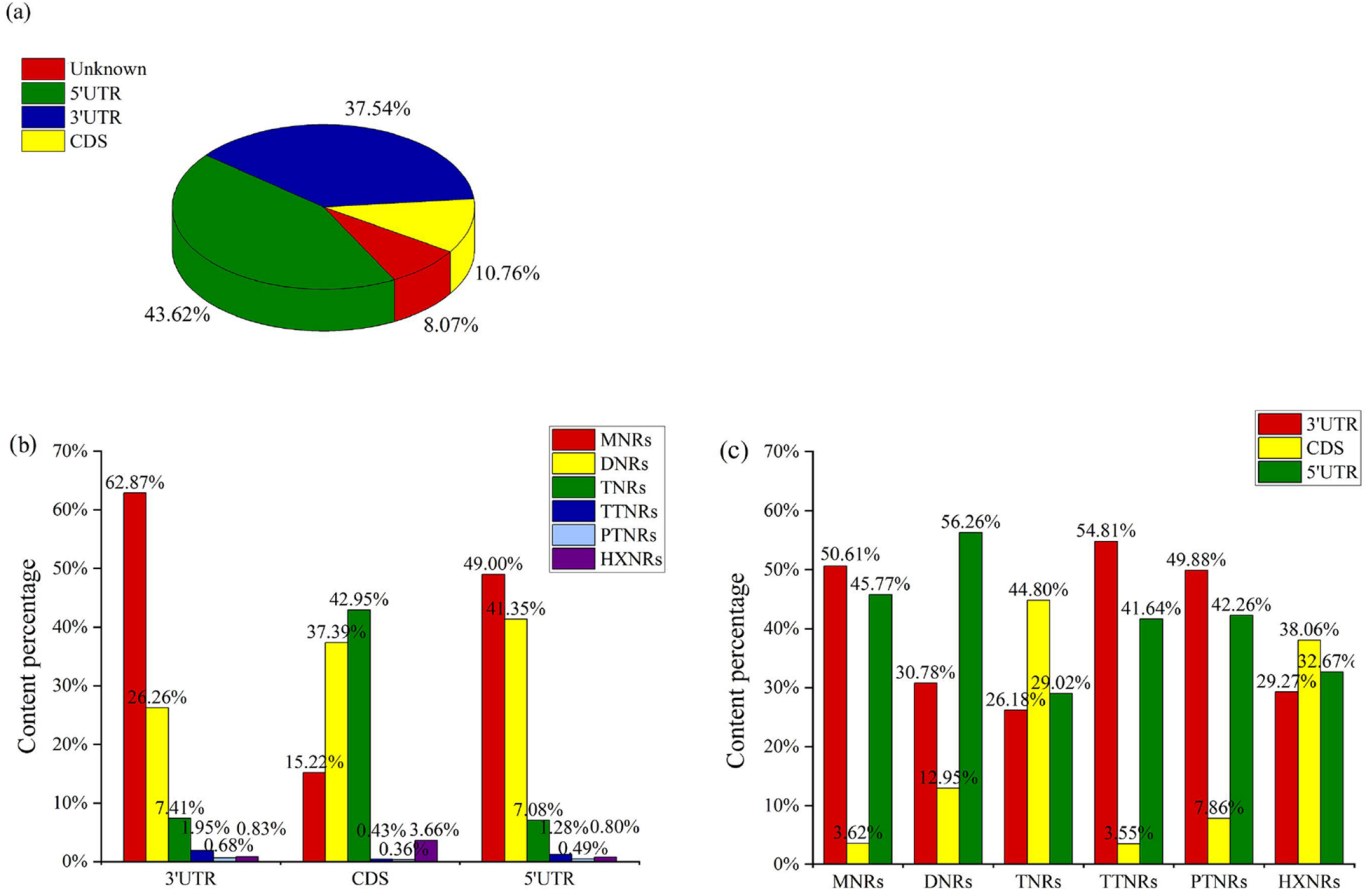
Distribution of SSRs in unigenes in the full-length transcriptome of *C. chekiangoleosa.* (**a**) The distribution of SSRs in unigenes. (**b**) The proportion of SSRs of different repetitive types distributed in the 3'UTR, 5'UTR and CDS regions. (**c**) The proportion of SSRs distributed in the 3'UTR, 5'UTR and CDS regions among different repetitive SSRs.

According to the statistics on the SSR distribution in unigenes in the *C. chekiangoleosa* full-length transcriptomic SSR database, the proportions of SSRs in the 5'UTR and 3'UTR were 43.62 and 37.54%, respectively, and only a small fraction of SSRs (10.76%) were distributed in the CDS region (Fig. 2a). Based on the statistical analysis of perfect SSRs located in CDS and UTR regions, the proportions of SSRs of each repeat type in the 3'UTR and 5'UTR presented the following order from high to low: mono-, di-, tri-, tetra-, hexa- and pentanucleotide (Fig. 2b). In the CDS region, trinucleotide repeats were the main type of SSR (42.95%), followed by dinucleotide repeats (37.39%), while pentanucleotide repeats were the least common, accounting for only 0.36% of the SSRs (Fig. 2b). The SSRs with mononucleotide, tetranucleotide and pentanucleotide repeats were mainly distributed in the 3'UTR, accounting for 50.61, 54.81 and 49.88% of the total SSRs, respectively (Fig. 2c). The SSRs with trinucleotide (44.80%) and hexanucleotide repeats (38.06%) were mainly distributed in the CDS region, and 56.26% of the dinucleotide repeats were distributed in the 5'UTR.

### Functional analysis and transcription factor prediction based on transcripts containing SSRs

**Figure 3:**
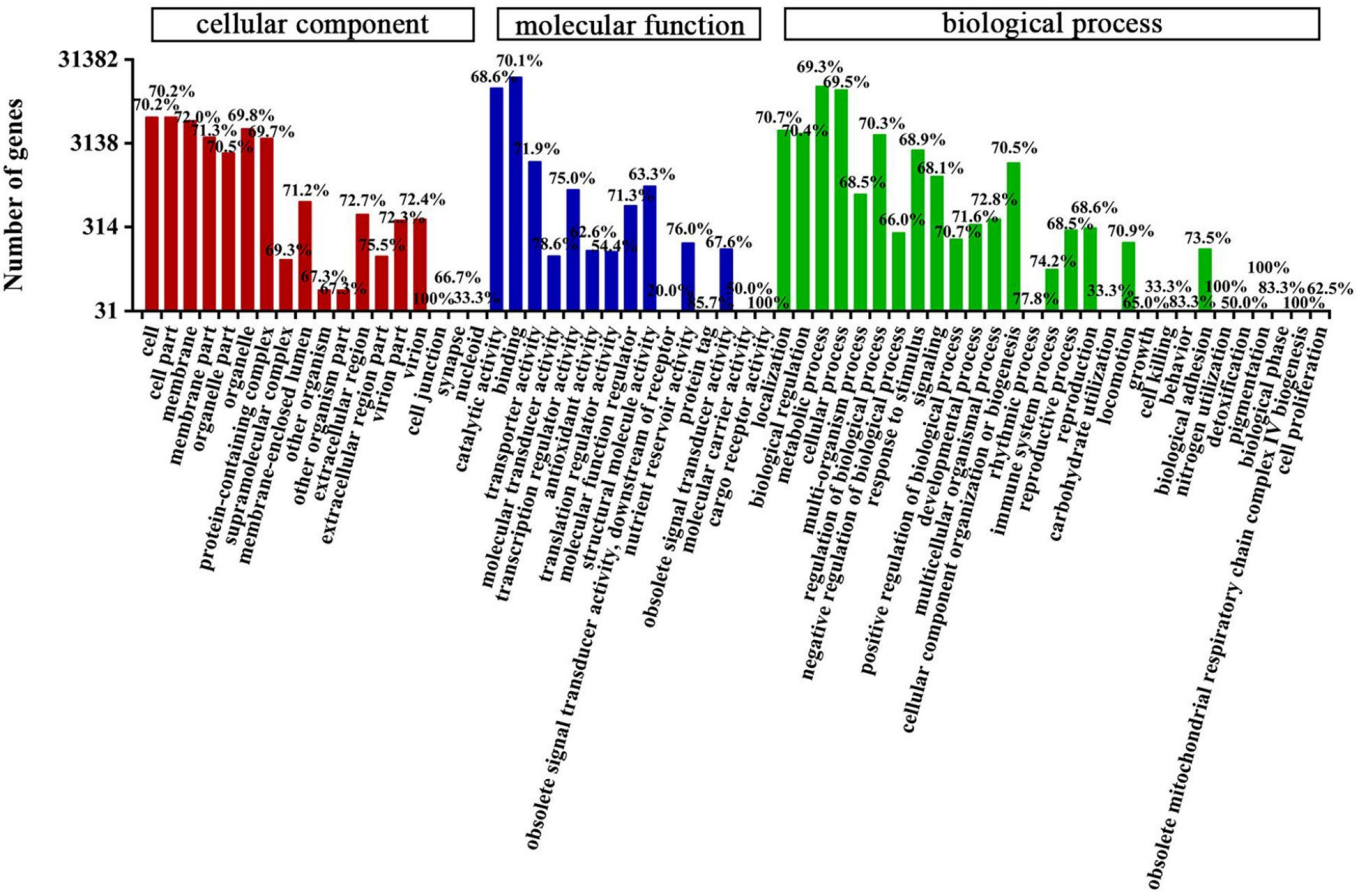
GO annotations of *C. chekiangoleosa* transcript sequences. The percentages above the bar chart indicate the proportion of transcripts containing SSRs among the annotated transcripts.

A total of 65,215 unigenes (48,323 containing SSRs) were compared with the GO and KEGG databases. The analysis revealed that the number of unigenes containing SSRs and the total number of unigenes showed a very significant correlation (P < 0.01) regarding the distribution ratio of the annotated GO functional groups and annotated KEGG metabolic pathways. There were 31,382 unigenes (69.93% containing SSRs) in the GO database that were annotated (Supplementary Table S2, Fig. 3). A total of 35,095 (70.64% containing SSRs), 38,455 (69.55%), and 49,670 (69.70%) unigenes were classified into the cellular component, molecular function and biological process categories, respectively. Within the cellular component category, cells and cell parts (6393 unigenes; 70.20% containing SSRs) constituted the largest group of unigenes, followed by membrane structure (5799; 72.03%), whereas the nucleoid (3; 33.33%) constituted the smallest group. In this category, the highest proportion of unigenes containing SSRs was associated with cell junctions (100.00%), and the lowest proportion was associated with the nucleoid (33.33%). Similarly, in the molecular function category, the unigenes involved in binding (19,147; 70.15%) constituted the largest group, and there were very few unigenes related to obsolete signal transmitter activity (5; 20.00%) or cargo receiver activity (2; 100.00%). The proportion of unigenes (20.0%) containing SSRs that were related to absolute signal transmitter activity was the lowest, while that related to cargo receiver activity was the highest (100%). Most unigenes involved in the biological process category were annotated to metabolic process (14,921; 69.33%) and cellular process (13,520; 69.55%). All unigenes annotated to nitrogen utilization, pigmentation and obsolete mitochondrial respiratory chain complex IV biogenesis groups contained SSRs. In the carbohydrate utilization and cell killing functional groups, only 33.3% of unigenes contained SSRs.

**Figure 4:**
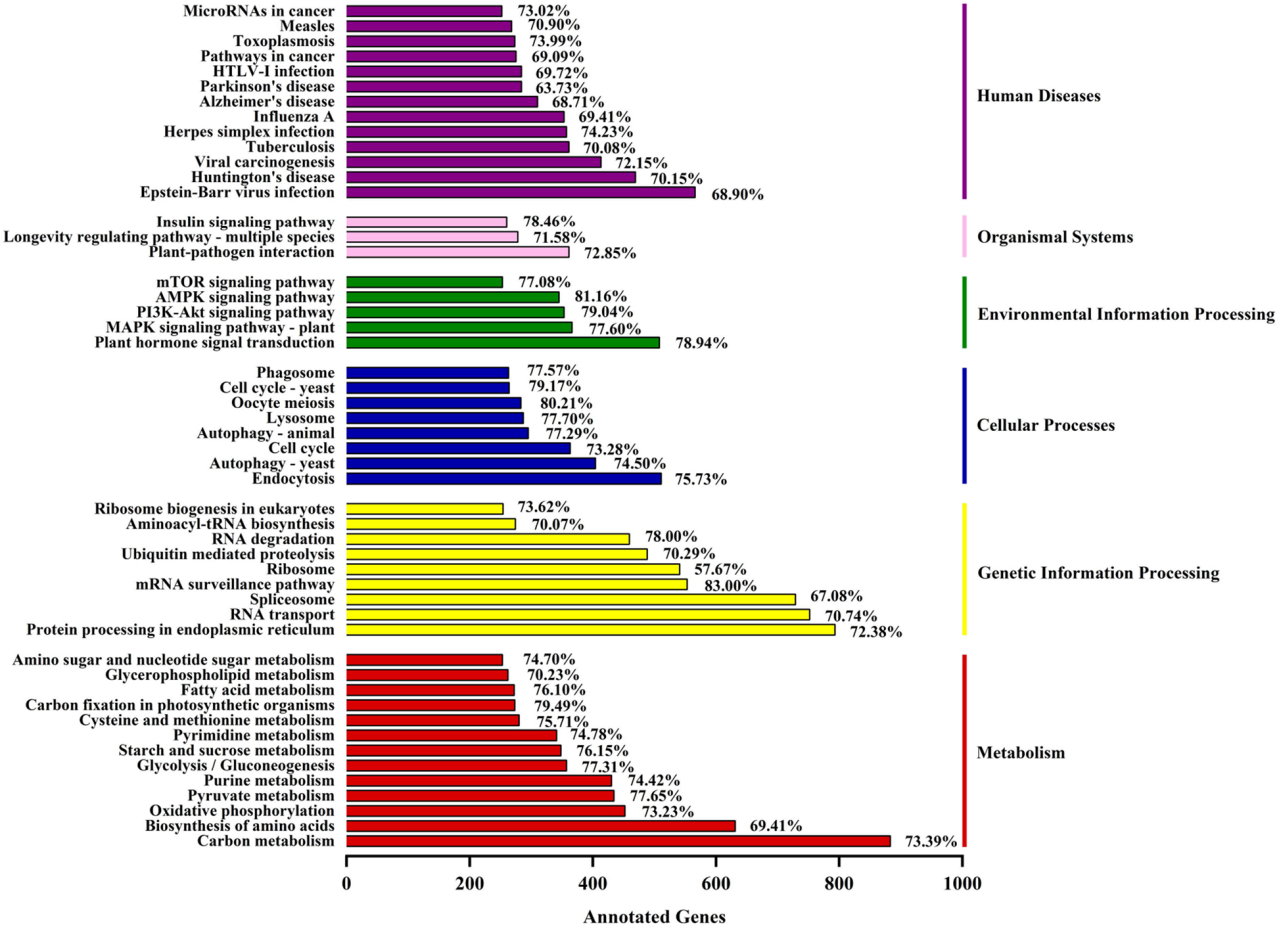
KEGG metabolic categories in the *C. chekiangoleosa* transcriptome. The percentages on the right side of the bar chart indicate the proportion of transcripts containing SSRs among the annotated transcripts.

**Figure 5:**
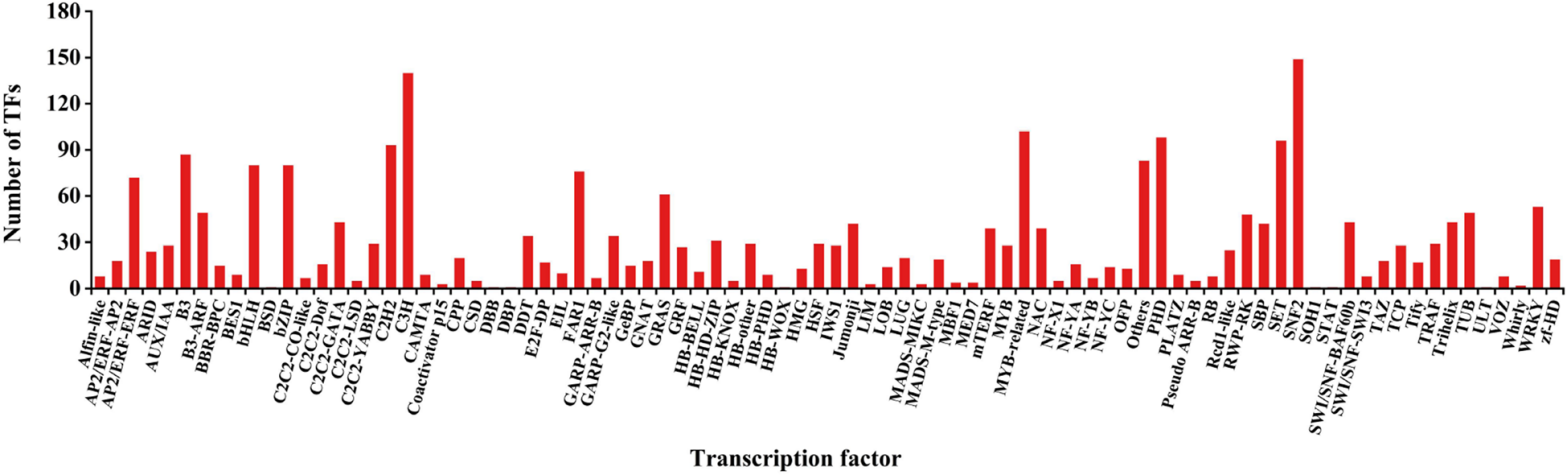
Analysis of the TFs in SSR-containing transcripts.

A total of 54,366 unigenes (72.63% containing SSRs) were annotated in the KEGG database; these unigenes were involved in 6 categories (metabolism, genetic information processing, cellular processes, environmental information processing, body systems and human diseases) and 357 metabolic pathways (Supplementary Table S3, Fig. 4). Most unigenes were related to metabolism (12,920), followed by human diseases (9212), and the fewest were related to cellular processes (4296). The proportions of unigenes containing SSRs involved in metabolism, genetic information processing, cellular processes, environmental information processing, biological systems and human diseases were 73.35, 70.72, 75.54, 75.63, 74.73, and 71.79%, respectively. There were four types of metabolic pathways related to oil: fatty acid metabolism (272 unigenes, 76.10% containing SSR); fatty acid biosynthesis (188; 77.66%); unsaturated fatty acid biosynthesis (100; 71.00%); and alpha-linolenic acid metabolism (107; 74.77%). In addition, some metabolic pathways were related to glycolysis (357; 77.31%), the phosphatidylinositol signaling system (212; 72.64%), plant hormone signal transduction (508; 78.94%), the MAPK signaling pathway (103; 64.08%), the AMPK signaling pathway (345; 81.16%) and the calcium signaling pathway (108; 71.30%) (Supplementary Table S3, Fig. 4). We predicted that 3091 unigenes encoded TFs, among which 74.60% also contained SSRs (Supplementary Tables S4a,b). These TFs were divided into 86 TF families, among which the main families were *SNF2* (149; 5.84%), *C3H* (140; 5.48%), *MYB-related* (102; 4.00%), *PHD* (98; 3.84%), *SET* (96; 3.76%) and *C2H2* (93; 3.64%) (Supplementary Table S4c, Fig. 5).

### SSR primer screening and polymorphism verification

**Figure 6:**
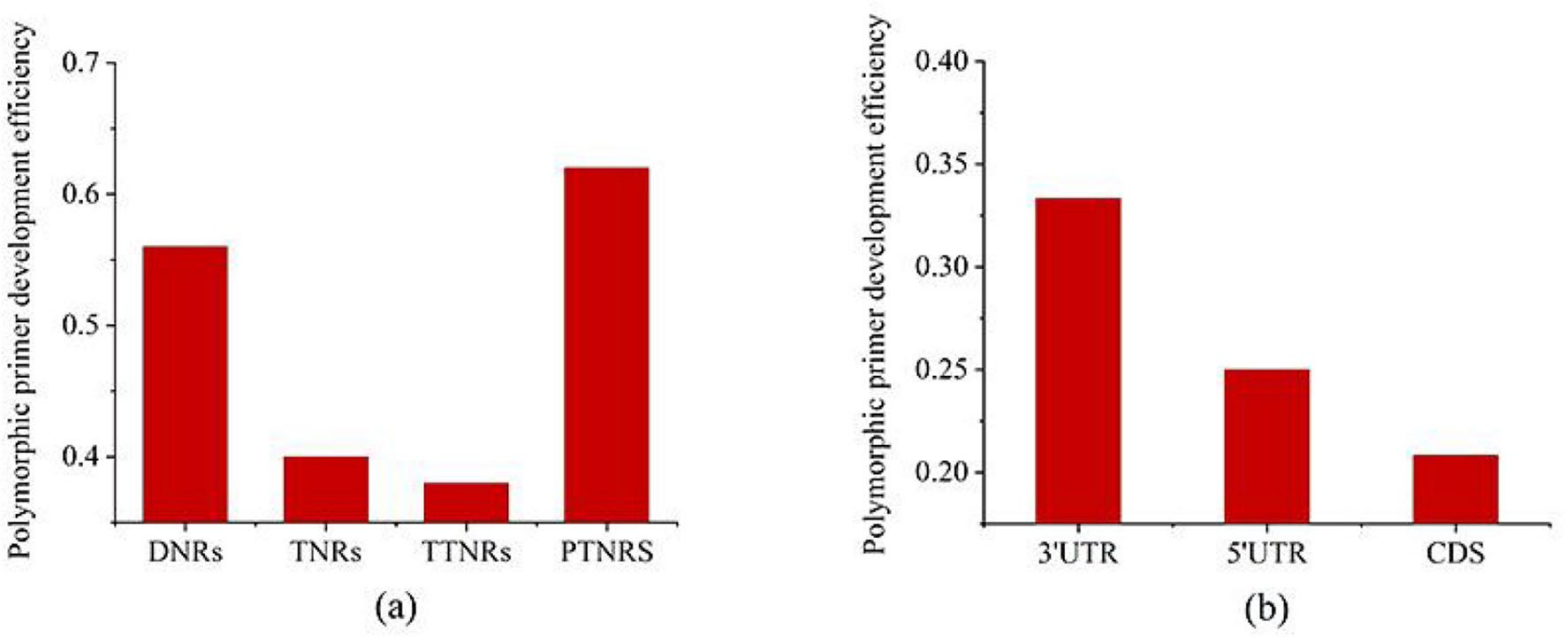
Development efficiency of polymorphic SSR primers. (**a**) Development efficiency of polymorphic primers for dinucleotide to pentanucleotide repeats. (**b**) Polymorphic primer development efficiency in UTR and CDS regions.

The PCR amplification results of different types of SSRs showed that there were 30 (60.00%), 34 (68.00%), 33 (66.00%) and 36 pairs (72.00%) of amplifiable primers for the di-, tri-, tetra-, pentanucleotide repeat types, respectively, while there were 28 (56.00%), 20 (40.00%), 19 (38.00%) and 31 pairs (62.00%) of polymorphic primers, and the proportions of polymorphic primers were 93.33%, 58.82%, 57.58% and 86.11%, respectively (Table 2, Fig. 6a). Among the amplifiable primers, the proportions of primers with a base length of ≥ 20 bp accounted for 73.33%, 29.41%, 57.58% and 86.11% of the primers. Finally, 580 pairs of SSR primers were counted. After screening, 300 pairs (51.72%) of primers were able to amplify clear bands, among which 155 pairs (26.72%) of polymorphic SSR primers were identified (Supplementary Table S5), and the total proportion of polymorphic primers was 51.67%. A total of 360 primer pairs targeting the 3'UTR (120 pairs), 5'UTR (120 pairs) and CDS (120 pairs) regions were randomly selected from the 580 synthesized pairs of SSR primers. The statistical results showed that the amplification efficiencies of the primers targeting the 3'UTR and 5'UTR were 62.50% and 54.17%, the development efficiencies of the polymorphic primers were 33.33% and 25.00%, and the proportions of polymorphic primers were 53.33% and 46.15%, respectively. The primer amplification efficiency, polymorphic primer development efficiency and proportion of polymorphic primers in the CDS region were 50.83%, 20.83% and 40.98%, respectively (Fig. 6b).

**Table 2:** Experimental results for di-, tri-, tetra- and pentanucleotide repeat SSR markers.

Repeat type	Primer development efficiency	Proportion of polymorphic primers		
		Overall	< 20 bp	≥ 20 bp
DNRs	60.00%	93.33%	20.00%	73.33%
TNRs	68.00%	58.82%	29.41%	29.41%
TTNRs	66.00%	57.58%	0	57.58%
PTNRs	72.00%	86.11%	0	86.11%

### Effectiveness of the SSR primers based on population analysis

**Figure 7:**
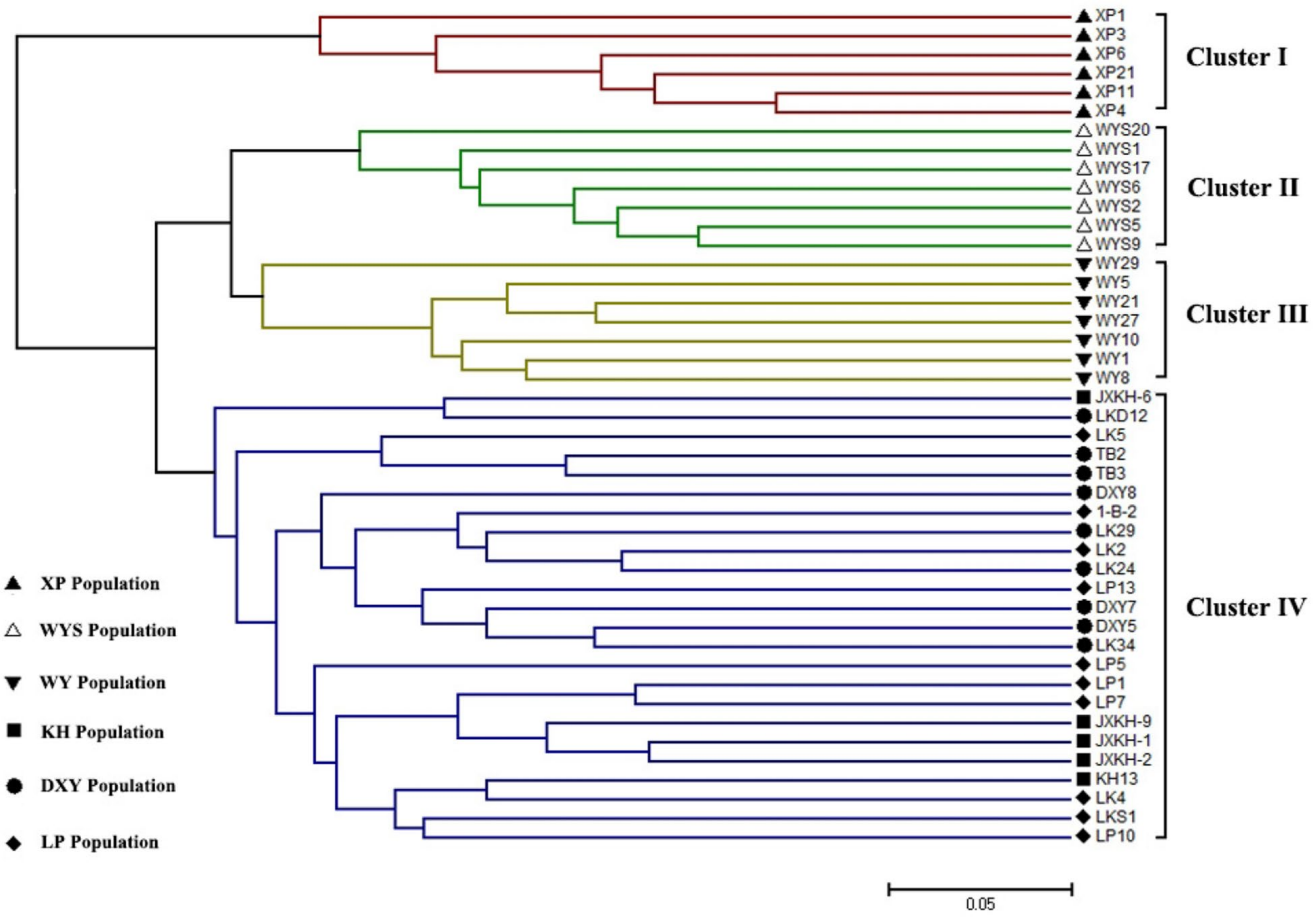
UPGMA cluster map of 44 *C. chekiangoleosa* individuals based on SSR markers.

We selected 44 samples of *C. chekiangoleosa* to further evaluate the polymorphisms in 27 pairs of primers. The test results showed that a total of 103 alleles were obtained, the number of alleles (*Na*) ranged from 2 to 7 per locus, and the average number of alleles was 4 (Table 3). The values of observed heterozygosity (*Ho*) and expected heterozygosity (*He*) ranged from 0 to 0.795 and 0.087 to 0.782, respectively, and the mean values were 0.402 and 0.585, respectively. The Leping (LP) and Xiapu (XP) populations showed the highest (0.504) and lowest (0.390) genetic diversity, respectively (Supplementary Table S6). The polymorphism information content (PIC) of the 27 SSR markers ranged from 0.083 to 0.748, with an average value of 0.528. Based on the UPGMA clustering method, 44 *C. chekiangoleosa* genotypes were clearly divided into four clusters (Fig. 7). All individuals in the XP, Wuyishan (WYS) and Wuyuan (WY) populations were grouped into cluster I, cluster II, and cluster III, respectively. The fourth cluster was mixed and included the three populations Kaihua (KH), Dexing (DXY) and LP, and there was almost no boundary between the DXY and LP populations.

**Table 3:** EST-SSR genetic diversity parameters of 44 *C. chekiangoleosa* individuals.

SSR loci	*Na*	*Ho*	*He*	PIC
CC_eSSR152	4	0.318	0.549	0.484
CC_eSSR165	4	0.500	0.671	0.600
CC_eSSR180	3	0.500	0.616	0.540
CC_eSSR284	6	0.500	0.730	0.685
CC_eSSR327	5	0.614	0.746	0.702
CC_eSSR369	4	0.318	0.709	0.653
CC_eSSR374	3	0.409	0.660	0.586
CC_eSSR393	5	0.364	0.782	0.748
CC_eSSR428	6	0.659	0.766	0.729
CC_eSSR437	4	0.795	0.688	0.632
CC_eSSR438	4	0.591	0.648	0.596
CC_eSSR442	4	0.614	0.681	0.624
CC_eSSR454	4	0.341	0.600	0.537
CC_eSSR460	2	0.000	0.201	0.181
CC_eSSR462	4	0.432	0.717	0.665
CC_eSSR472	4	0.341	0.621	0.559
CC_eSSR477	3	0.500	0.615	0.533
CC_eSSR486	6	0.500	0.758	0.723
CC_eSSR532	3	0.364	0.585	0.496
CC_eSSR537	2	0.136	0.325	0.272
CC_eSSR546	2	0.045	0.087	0.083
CC_eSSR570	2	0.136	0.499	0.374
CC_eSSR619	2	0.409	0.499	0.374
CC_eSSR628	2	0.045	0.397	0.318
CC_eSSR676	7	0.659	0.733	0.690
CC_eSSR680	6	0.636	0.753	0.719
CC_eSSR692	2	0.136	0.165	0.152
Mean	4	0.402	0.585	0.528

## Discussion

### SSR distribution characteristics

Based on consistent sequencing methods and SSR retrieval criteria, the SSR occurrence frequency (74.03%) in the full-length transcriptome of *C. chekiangoleosa* was close to that of *Rhododendron lapponicum* (61.23%)^30^ but was substantially higher than those reported for *P. wulianensis* (37.95%)^26^ and *Madhuca pasquieri* (30.86%)^31^. Among woody plants, both *C. chekiangoleosa* and *R. lapponicum* have large-scale full-length transcriptomes (large number of unigenes and long sequences) and show a high frequency of SSR occurrence^26,30,31^. Therefore, we speculate that the length of full-length unigene transcripts and the overall sequence quality may influence the observed occurrence frequency of SSRs. The main repeat types of SSRs in the full-length transcriptome of *C. chekiangoleosa* were dinucleotides and trinucleotides (mononucleotide repeats were not considered), which was similar to the findings of a study of a closely related species of *Camellia. sinensis*^32^. This result was also consistent with the results found for other species, such as *Persea americana* Mill.^33^, *Styrax japonicus*^34^, and *Paulownia catalpifolia*^35^. The differences in the transcriptomic information and basic SSR characteristics between *C. chekiangoleosa* and other species might be due to species specificity, sequencing technology, reference genome quality and other factors. Based on Illumina sequencing technology, Li et al. found that the main repeat types of SSRs (occurrence frequency of 26.75%, average span of 2.33 kb) in the transcriptome of *C. oleifera* were dinucleotide (AG/CT), mononucleotide (A/T) and trinucleotide (AAG/CTT) repeats^14^. Based on Roche 454 sequencing analysis, the average SSR length in the *C. chekiangoleosa* genome was 1.85 kb, and dinucleotides (AG), mononucleotides (A/T), pentanucleotides (AAAAT) and trinucleotides (AAT) were the main repeat types^36^. The main repeat types of transcriptomic SSRs in *C. chekiangoleosa* (5.50%, 6.25 kb), *C. oleifera* and *Camellia. brevistyla* were dinucleotides (AG), trinucleotides (AAG) and hexanucleotides, whereas the genomic SSRs of *C. oleifera* were dominated by dinucleotide (AG), trinucleotide (AAT) and tetranucleotide repeat types^18^. Based on the above reports, dinucleotides (AG) and trinucleotides (AAG) are the main repeat types in the full-length transcriptome of *C. chekiangoleosa*, which is consistent with the results obtained for related species transcriptomes^14,18,32^. However, our finding was slightly different from the result that AAT was the main type of trinucleotide motif in the genome^18,36^. Therefore, in the transcriptome of *Camellia* plants, AG may be the main repetitive motif of dinucleotide SSRs.

AAG may be the main repetitive motif of trinucleotide SSRs. In this study, the main repetitive motifs identified among the full-length transcriptome SSRs of *C. chekiangoleosa* were rich in A/T, while the G/C content was very low, which may be because GC motifs are included in certain amino acid sequences and are related to specific functions^37^.

Species containing a large number of short repeat motifs show high levels of evolution^38^. In this study, mononucleotides, dinucleotides and trinucleotides were the main SSR repeat types, indicating that *C. chekiangoleosa* might exhibit a high mutation rate or a relatively high level of evolution. SSR sequences are an important factor in the genetic variation available during the process of species evolution^39^. The basic characteristics of SSRs in the full-length transcriptome of *C. chekiangoleosa* will provide some clues regarding the genetic evolution of *Camellia* plants.

### SSR statistical analysis of full-length transcripts of functionally annotated unigenes

The longer the read length of the full-length transcripts obtained based on the PacBio platform, the more comprehensive the gene information and the higher the annotation efficiency of functional genes will be^24,40^. Compared with the full-length transcriptomes of *C. sinensis*^32^ (2469 bp), *C. oleifera* (2114 bp)^41^ and *R. lapponicum* L (2509 bp)^30^, the obtained unigenes of the full-length transcriptome of *C. chekiangoleosa* exhibited a longer average read length (2887.87 bp). Therefore, the transcript annotation information obtained in this study was more comprehensive and reliable.

On the basis of the statistical analysis, it was noted that there was a significant positive correlation between the number of unigenes and the number of SSR-containing unigenes in each GO and KEGG functional classification. However, the proportion of SSR-containing unigenes in each functional classification was not consistent with the number of unigenes; that is, the proportion of SSR-containing unigenes in the functional classification with the most annotations was not the largest. Among the GO annotations, the SSR-containing unigenes were mainly annotated to the binding and metabolic process categories, while the largest proportions of SSR-containing unigenes were associated with cell junctions, cargo receptor activity, and nitrogen utilization. The categories annotated with the most SSR-containing unigenes in the KEGG database were carbon metabolism, protein processing in the endoplasmic reticulum, and RNA transport. However, the largest proportions of SSR-containing unigenes were annotated to categories such as geraniol degradation and naphthalene. Phosphatidylinositol (PI) is associated with the osmotic regulation and defensive responses of plants^42^. In addition, glycolysis and alpha-linolenic acid metabolism directly affect the yield and quality of Oil-camellia^43^. Based on the KEGG analysis, we identified glycolysis, phosphatidylinositol signaling system, and fatty acid metabolism pathways, as well as other pathways related to the oil content of seed kernels; the alpha-linolenic acid metabolism pathway related to the quality of oil; and functional annotation information related to stress resistance pathways such as plant hormone signal transduction and the MAPK signaling pathway. TFs are key regulators of gene expression, and *AP2/ERF* TFs play an important role in fatty acid production^44^. Gong et al. found that multiple TFs were significantly correlated with oil content in the full-length transcriptome of *C. oleifera*, among which *MYB* TFs played a negative regulatory role in oil accumulation in seeds, *AP2/ERF* TFs were significantly correlated with a high oil content, and *bZIP* TFs contributed to the transcriptional regulation of genes involved in oil synthesis in *C. oleifera* seeds^41^. These TFs involved in oil synthesis in *C. oleifera* were also predicted in the full-length transcriptome sequence of *C. chekiangoleosa*, and SSR sites were detected simultaneously in the unigene sequences of *AP2/ERF* (90), *MYB* (130) and *bZIP* (80) TFs. The GO functional annotation, KEGG metabolic pathway and SSR site information of the full-length transcriptome of *C. chekiangoleosa* provide a source for the development of functional markers, which provide a basis for important agronomic traits and molecular marker-assisted selection.

### Development strategy and verification of polymorphic SSR markers

We expected to synthesize 600 pairs of SSR primers. Some primers did not successfully amplify regions of interest due to alternative splicing and other factors. In total, 580 pairs of primers were selected for analysis. The amplification efficiency of SSR primers (51.72%) and the development efficiency of polymorphic primers (26.72%) in the full-length transcriptome of *C. chekiangoleosa* were higher than those in the *C. oleifera* transcriptome (48.95%; 13.99%)^14^ and the *P. americana* transcriptome (31%; 16%)^33^ but lower than those in a previous study on the *C. chekiangoleosa* genome (65.56%; 31.9%)^19^. Compared with other studies aimed at SSR marker development based on a full-length transcriptome, the SSR primer amplification efficiency and polymorphic primer development efficiency achieved in *C. chekiangoleosa* were lower than those reported in sugarcane (79.1%; 32.6%)^27^, but the polymorphic primer development efficiency was substantially higher than that in *P. wulianensis* (18%)^26^.

The mutation pattern of SSRs is extremely complex and includes mutations occurring between sites and mutations that control the evolution of a single SSR locus^45^. To further study the mutation pattern of SSRs and develop a set of effective SSR polymorphic primer development strategies, this study focused on analyzing the length variation of SSRs in the full-length transcriptome of *C. chekiangoleosa* and analyzed the distribution of di-, tri-, tetra-, and pentanucleotide repeats and polymorphic markers in unigene sequences. The SSR length variation rule in the full-length transcriptome of *C. chekiangoleosa* was as follows: except for the hexanucleotide repeats, the theoretical polymorphism of the SSRs was inversely proportional to the base length of the repeat unit. This result was consistent with the research results reported for the *C. chekiangoleosa* transcriptome and genome^18,19^, as well as the hypothesis that the degree of variation of dinucleotide repeats is higher than that of trinucleotide repeats put forth by Ashworth et al.^46^ However, our experimental results showed that the development of polymorphic SSR primers was most efficient for pentanucleotide repeats, followed by di-, tri- and tetranucleotide repeats; the proportion of polymorphic primers showed the following order: di-, penta-, tri- and tetranucleotide repeats. Previous studies have shown that the polymorphic level of SSRs positively correlates with their sequence length: SSRs with lengths ≥ 20 bp show higher polymorphism than SSRs with lengths < 20 bp^29,47^. Shi et al. found that long repeats (≥ 20 bp) of trinucleotides, tetranucleotides and pentanucleotides in the genome of *C. chekiangoleosa* were more variable than short repeats^19^. Based on our experimental results, it is possible that the degree of variation of pentanucleotide repeats mainly depends on the variability of long repeats. Whether our experimental conclusion represents a universal law of *C. chekiangoleosa* SSRs needs to be further explored.

Studying the distribution tendencies of SSRs in gene regions can lay a foundation for the subsequent development and use of SSR markers. SSRs in the whole genomes of *Arabidopsis thaliana* and rice are concentrated in UTRs (5′UTRs show the highest distribution density)^48^. A large number of SSR loci were found to be distributed in UTRs (5'UTR > 3'UTR), and a small number were located in CDS regions, which was consistent with the information on the distribution of SSR loci in the whole genomes of the model plants *A. thaliana* and rice^48^. This finding differs slightly from the results obtained for *Elaeagnus mollis* Diels^49^ (3'UTR > 5'UTR) but is generally consistent with the views that SSRs are more frequently distributed in transcribed regions and that UTRs represent their main distribution area^50^. In the full-length transcriptome of *C. chekiangoleosa*, the CDS region was dominated by trinucleotide repeats, and the UTR was dominated by dinucleotide repeats (except for single-nucleotide repeats). This finding is consistent with the report of Wen et al. involving *C. chekiangoleosa*^18^. Nontriple repeats can cause coding region frameshift mutations, and a large number of nucleotides in CDS may avoid the occurrence of mutations to the greatest extent possible^51^. SSRs in different regions have different functions, and their polymorphism levels also differ. SSRs located in the 5'UTR function in gene expression regulation, and when the SSRs in the CDS region are mutated, they impact protein translation^18,52,53^. Therefore, the SSRs located in the 5'UTR are more conserved than those in the 3'UTR, and the CDS region should be more conserved than the UTRs. Through experiments, we found that the development efficiency and proportion of polymorphic SSR markers in the full-length transcriptome of *C. chekiangoleosa* presented the following order: 3'UTR > 5'UTR > CDS region. This result verified the above perspective.

### Detection of the effectiveness of *C. chekiangoleosa* SSR markers

The PIC is an index reflecting the polymorphism of SSR primers. The larger the PIC value is, the higher the polymorphism of the primer. When PIC < 0.25, this locus shows low polymorphism; when 0.25 > PIC < 0.5, it shows moderate polymorphism, and PIC > 0.5 shows high polymorphism^54^. In this study, there were 16 SSR markers with a PIC > 0.5, 6 markers with values between 0.25 and 0.5, and only 3 markers with a PIC < 0.25. The average PIC value of the 27 SSR markers was 0.528, indicating that most of the SSR markers developed here have high polymorphism. The cluster diagram clustered 44 tested samples into four groups, and the individuals in each group were well divided according to origin. This result shows that the SSR markers we developed can distinguish populations well. The provenance of the LP population comes from the DXY population. Their genetic backgrounds are very similar, and the two populations were clustered together. Moreover, the KH population grouped with the DXY and LP populations, which is presumably due to the closer spatial distance between the KH and DXY populations. The clustering results further reflect that the genetic distance of each population of *C. chekiangoleosa* shows a certain linear relationship with the spatial distance of the place of production.

SSR markers are one of the most effective molecular markers for detecting plant genetic diversity^55^ and have been used in research on the genetic diversity and population structure of oil-camellia plants. In these reports, *C. oleifera*^56^ (0.79) and *Camellia nitidissima*^57^ (0.546) all showed medium or high He values. In this study, the six different geographic populations of *C. chekiangoleosa* also showed a high level of genetic diversity (0.585), which is similar to the genetic diversity of *C. nitidissima*^57^ (0.546) and slightly higher than that of *Camellia reticulata*^58^ (0.457). *C. chekiangoleosa* is a highly self-incompatible cross-pollinated species with high heterozygosity, which contributes to its high genetic diversity.

## Conclusions

This study identified 97,510 SSR sites in 65,215 unigene sequences, and the development efficiency of polymorphic SSR markers was 26.72%. We obtained a large number of SSR-containing unigene sequences involved in metabolic processes, important biosynthesis pathways and signal transduction mechanisms based on GO and KEGG annotations and TF prediction. Experiments have verified the efficient development of polymorphic SSR markers of different repeat types in the following order: pentanucleotides > dinucleotides > trinucleotides > tetranucleotides. The developmental efficiency of polymorphic SSR markers in different regions was in the order of 3'UTR area > 5'UTR area > CDS area. In addition, we used 44 excellent clones to further evaluate the applicability of 27 SSR markers in the study of the population genetic diversity of *C. chekiangoleosa.* These data will help to efficiently develop functional SSR markers for *C. chekiangoleosa* and lay a foundation for subsequent research on population genetic diversity, functional gene mining, and marker‒trait association analysis.

## Materials and methods

### Plant material and DNA extraction

The 44 outstanding clones used in the polymorphism evaluation test of the SSR markers^59^ were derived from WY, DXY, LP (Dexing provenance plantation above 15a), XP, WYS and KH, for a total of 6 *C. chekiangoleosa* production areas (Supplementary Table S1). Sixteen individuals of *C. chekiangoleosa* (Wuyuan, Jiangxi Province, China, longitude 118.06°E, latitude 29.24°N, and altitude 580–820 m) with different genotypes were used as DNA templates to screen the effectiveness of the primers. Plant materials were collected in compliance with the institutional, national, and international guidelines and legislation. Young leaves were taken and stored at − 80 °C until the experiment. DNA was extracted via the modified cetyltrimethylammonium bromide (CTAB) method^60^, and the concentration and purity of the DNA were detected with a NanoDrop2000 system (Thermo Scientific, USA), after which the DNA was stored at − 20 °C for later use.

### Source of transcriptome sequences and SSR mining

The sequences used for SSR locus mining were derived from the full-length transcriptome data of *C. chekiangoleosa*. The full-length transcriptome based on the PacBio sequencing platform was corrected by using the second-generation transcriptome. Finally, full-length nonchimeric reads (FLNC) were obtained (SRA accession PRJNA753883)^28^. The materials employed for sequencing were seeds at different developmental stages obtained from excellent individual plants at the experimental base of the Jiangxi Academy of Forestry, China. MISA software^61^ (version: 1.0) was used to retrieve the SSR sites in the full-length transcriptome of *C. chekiangoleosa*, after which an SSR database was established. The search criteria were as follows: repeat numbers of mono-, di-, tri-, tetra-, penta-, and hexanucleotide SSRs were greater than or equal to 10, 6, 5, 5, 5 and 5, respectively.

### Distribution of SSR loci and associated unigene annotation

The unigenes containing SSRs were compared with the GO and KEGG databases^62^ (www.kegg.jp/kegg/kegg1.html) via BLAST software (version 2.2.26)^63^, and TFs were predicted by using iTAK software^64^ (Version 1.7a: https://github.com/kentnf/iTAK/). The frequency of occurrence was calculated as the number of SSR-containing unigene sequences divided by the total number of unigene sequences. The average span referred to the distance between each SSR site and was calculated as the total sequence length divided by the total number of SSRs. When counting repeat motif types, all possible + 1 frameshift motifs and their complementary sequences were regarded as the same motif type. For example, the sum of the number of occurrences of four dinucleotide repeat motifs, AC, CA, TG and GT, was regarded as the number of occurrences of the dinucleotide repeat motif AC^18^. The distribution of SSRs and the composition of 1–6 nucleotide repeats in the CDS and UTR were measured.

### Design and amplification of SSR primers

A total of 580 pairs of SSR primers were randomly developed based on the full-length transcriptomic data of *C. chekiangoleosa*, and 200 pairs of dinucleotide repeats (50 pairs), trinucleotide repeats (50 pairs), tetranucleotide repeats (50 pairs) and pentanucleotide repeats (50 pairs) were randomly selected for polymorphism detection. The development efficiency of the primers (the proportion of amplifiable primers among the total primers), the amplification efficiency of polymorphic primers (the proportion of polymorphic primers among the total primers) and the proportion of polymorphic primers (the proportion of polymorphic primers among the amplifiable primers) were quantified. SSR primers were designed in batches using Primer 3.0. To obtain easily amplified SSR primers, primers were designed based on the following criteria: the length of primers was 18–22 bp; the predicted PCR product size was 100–300 bp; the annealing temperature ranged from 57 °C to 60 °C; and the (G + C) content was 40–65%. The 10-µL SSR-PCR amplification system included the following components: 1 µL of 10 × buffer; 1 µL of Mg^2+^ (25 mmol/L); 1 µL of dNTPs (10 mmol/L); 0.4 µL of primer-F (10 µmol/L); 0.4 µL of primer-R (10 µmol/L); 0.1 µL of Taq enzyme (5 U/µL, Takara); 0.5 µL of DNA template (100 ng/µL); and 5.6 µL of ddH_2_O. SSR-PCR was performed under the following conditions: predenaturation at 94 °C for 5 min; denaturation at 94 °C for 30 s, annealing at 57–60 °C (depending on the primers) for 30 s, and extension at 72 °C for 30 s for 25 cycles; a final extension at 72 °C for 1 min; and storage at 4 °C. The PCR amplification products were detected using 8% polyacrylamide gel electrophoresis. Images and records of the gel were obtained after silver staining.

### Population detection and evaluation of SSR polymorphisms

We selected 27 markers with good polymorphism from the 155 polymorphic SSR markers obtained; we used 44 *C. chekiangoleosa* genotypes to evaluate the polymorphism of the markers and performed cluster analysis simultaneously. The number of alleles (Na), observed heterozygosity (Ho), expected heterozygosity (He) and polymorphism information content (PIC) were obtained by Power Marker V3.25 software^65^. The cluster analysis was carried out according to the unweighted pair-group method using the arithmetic average (UPGMA), and the cluster diagram was generated by MEGA 5.0 software^66^.

## Supplementary Information

Supplementary Information 1.

Supplementary Information 2.

## Data Availability

The full-length transcriptome raw data of *C. chekiangoleosa* in the study are accessible at NCBI under bioproject (PRJNA753883).
