# Supplementary material for: Microsatellite analysis and polymorphic marker development based on the full-length transcriptome of Camellia chekiangoleosa
Source: Sci Rep. 2022 Nov 7;12:18906. doi: 10.1038/s41598-022-23333-3 (PMC9640616; doi:10.1038/s41598-022-23333-3)
Supplement: Supplementary file 1 — Supplementary Information 1. [file 41598_2022_23333_MOESM1_ESM.pdf]

Supplementary Information

(Supplementary Figure S1, FigureS2)

(Supplementary Table S4a, Table S4c, Table S7)

Microsatellite analysis and polymorphic marker development based on the full-length transcriptome of *Camellia chekiangoleosa*

Qianqian Tian[1,2+] Bin Huang[1+] Jianjian Huang[1] Bo Wang[1] Le Dong[1] Xin Yin[1] Chun Gong[1] Qiang Wen[1*]

1 Jiangxi Provincial Key Laboratory of Camellia Germplasm Conservation and Utilization, Jiangxi Academy of Forestry, Nanchang 330047, China

2 College of Forestry, Jiangxi Agricultural University, Nanchang 330045, China

+ Co-first author, these authors contributed equally to this work

*the corresponding author: jxwenqiang@aliyun.com

Supplementary Material (Figure S1, Figure S2)

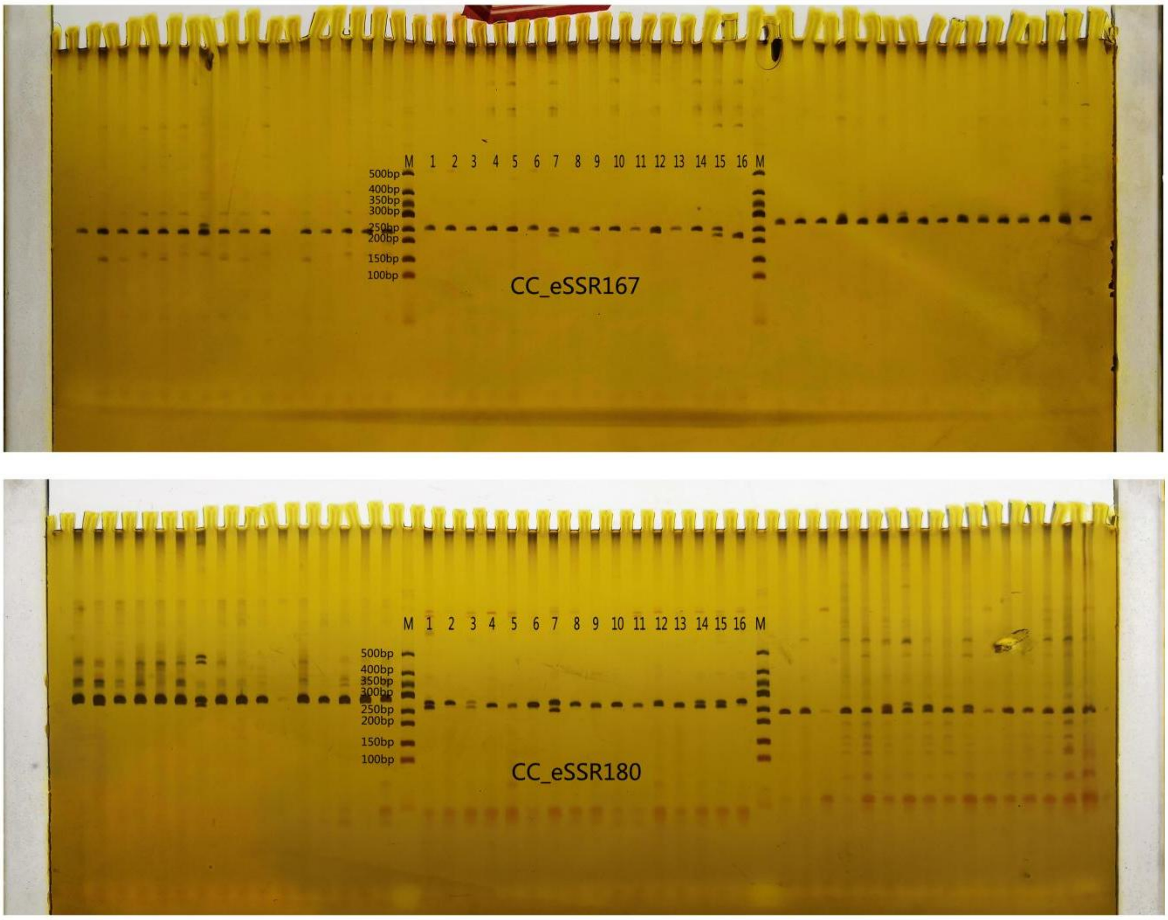

Figure S1. Amplification results of 16 *C. chekiangoleosa* samples with primers CC_eSSR167 and CC_eSSR180.

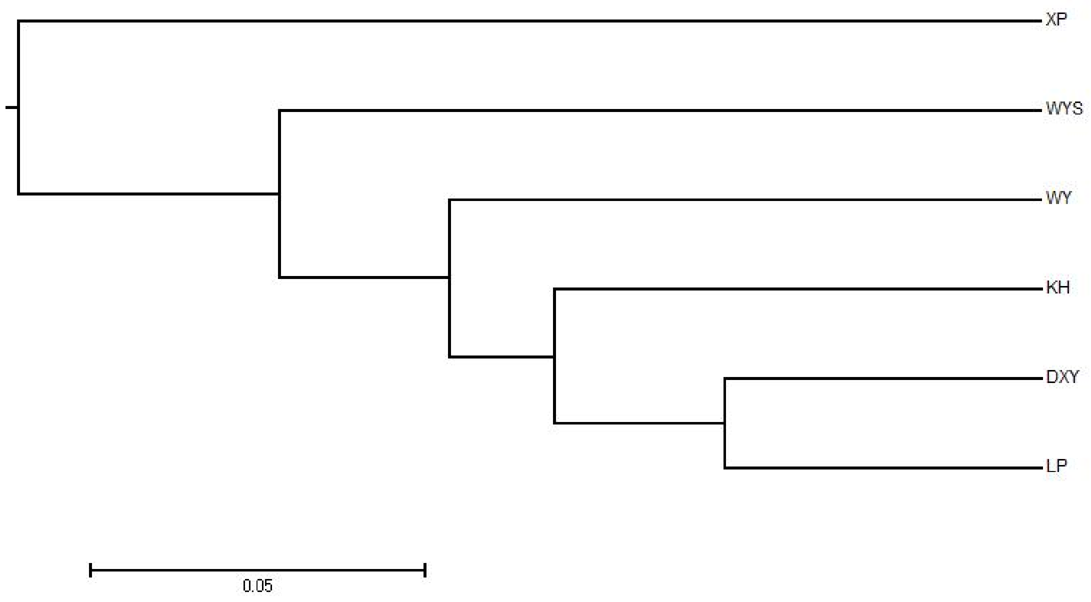

Figure S2. UPGMA cluster map of 6 *C. chekiangoleosa* populations based on SSR markers.

Supplementary Material (Table S4a, Table S4c, Table S7)

Table S4a. The basic features of SSR in TF-containing transcripts.

Total number of sequences examined/TF containing sequences	3091
Total number of identified SSRs	5688
Number of SSR containing sequences	2306
Number of sequences containing more than 1 SSR	1405
Number of SSRs present in compound formation	1162

Table S4c. Analysis of transcription factors in SSR-containing transcripts.

Family	Number	percentage	Family	Number	percentage
Alfin-like	8	0.31%	IWS1	28	1.10%
AP2/ERF-AP2	18	0.71%	Jumonji	42	1.65%
AP2/ERF-ERF	72	2.82%	LIM	3	0.12%
ARID	24	0.94%	LOB	14	0.55%
AUX/IAA	28	1.10%	LUG	20	0.78%
B3	87	3.41%	MADS-MIKC	3	0.12%
B3-ARF	49	1.92%	MADS-M-type	19	0.74%
BBR-BPC	15	0.59%	MBF1	4	0.16%
BES1	9	0.35%	MED7	4	0.16%
bHLH	80	3.13%	mTERF	39	1.53%
BSD	1	0.04%	MYB	28	1.10%
bZIP	80	3.13%	MYB-related	102	4.00%
C2C2-CO-like	7	0.27%	NAC	39	1.53%
C2C2-Dof	16	0.63%	NF-X1	5	0.20%
C2C2-GATA	43	1.68%	NF-YA	16	0.63%
C2C2-LSD	5	0.20%	NF-YB	7	0.27%
C2C2-YABBY	29	1.14%	NF-YC	14	0.55%
C2H2	93	3.64%	OFP	13	0.51%
C3H	140	5.48%	Others	83	3.25%
CAMTA	9	0.35%	PHD	98	3.84%
Coactivator p15	3	0.12%	PLATZ	9	0.35%
CPP	20	0.78%	Pseudo ARR-B	5	0.20%
CSD	5	0.20%	RB	8	0.31%
DBB	1	0.04%	Rcd1-like	25	0.98%
DBP	1	0.04%	RWP-RK	48	1.88%
DDT	34	1.33%	SBP	42	1.65%
E2F-DP	17	0.67%	SET	96	3.76%
EIL	10	0.39%	SNF2	149	5.84%
FAR1	76	2.98%	SOH1	1	0.04%
GARP-ARR-B	7	0.27%	STAT	1	0.04%
GARP-G2-like	34	1.33%	SWI/SNF-BAF60b	43	1.68%
GeBP	15	0.59%	SWI/SNF-SWI3	8	0.31%
GNAT	18	0.71%	TAZ	18	0.71%
GRAS	61	2.39%	TCP	28	1.10%
GRF	27	1.06%	Tify	17	0.67%
HB-BELL	11	0.43%	TRAF	29	1.14%
HB-HD-ZIP	31	1.21%	Trihelix	43	1.68%
HB-KNOX	5	0.20%	TUB	49	1.92%
HB-other	29	1.14%	ULT	1	0.04%
HB-PHD	9	0.35%	VOZ	8	0.31%
HB-WOX	1	0.04%	Whirly	2	0.08%

HMG	13	0.51%	WRKY	53	2.08%
HSF	29	1.14%	zf-HD	19	0.74%

Table S7. Information of 44 *C. chekiangoleosa* samples used in this study.

Pop.	Samples	Location	Latitude(°N)	Longitude(°E)	Altitude(m)
WY	WY5<br>WY8<br>WY10<br>WY21<br>WY27<br>WY29<br>WY1	Wuyuan County, Jiangxi Prov.	29°32′	118°09′	550
DXY	DXY5<br>DXY7<br>DXY8<br>LKD12<br>LK24<br>LK29<br>LK34<br>TB2<br>TB3	Dexing City, Jiangxi Prov.	29°27′	118°08′	720
LP	LP1<br>LP5<br>LP7<br>LP10<br>LP13<br>LKS1<br>LK2<br>LK4<br>LK5<br>1-B-2	Leping City, Jiangxi Prov.	28°92′	117°31′	60
XP	XP1<br>XP3<br>XP4<br>XP6<br>XP11<br>XP21	Xiapu County, Fujian Prov.	27°04′	120°02′	680
WYS	WYS1<br>WYS2<br>WYS5<br>WYS6<br>WYS9	Mount Wuyi, Fujian Prov.	27°73′	117°64′	1190

	WYS17<br>WYS20				
KH	JXKH-1<br>JXKH-2<br>JXKH-6<br>JXKH-9<br>KH13	Kaihua County, Zhejiang Prov.	29°27′	118°09′	590

Pop- populations; Prov- province.
